# Brown fat thermogenesis and cold adaptation in humans

**DOI:** 10.1186/s40101-025-00391-w

**Published:** 2025-04-21

**Authors:** Takeshi Yoneshiro, Mami Matsushita, Juro Sakai, Masayuki Saito

**Affiliations:** 1 Division of Molecular Physiology and Metabolism, Tohoku University Graduate School of Medicine, Sendai, Miyagi 980 - 8575 Japan; 2 Department of Nutrition, School of Nursing and Nutrition, Tenshi College, Sapporo, Hokkaido 065 - 0013 Japan; 3 Laboratory of Biochemistry, Faculty of Veterinary Medicine, Hokkaido University, Sapporo, Hokkaido 060 - 0818 Japan

## Abstract

Brown adipose tissue (BAT) is a site of non-shivering thermogenesis (NST) in mammals. Since the rediscovery of BAT in adult humans, there has been a remarkable advance in human BAT researches, revealing the significant roles of this thermogenic tissue in cold-induced NST and cold adaptation. Cold stress influences BAT in various time spans: acute cold exposure promptly activates BAT to induce NST, which contributes to immediate maintenance of body temperature. Prolonged cold exposure recruits BAT, resulting in increased capacity of NST and improved cold tolerance. Such BAT adaptation not only occurs in the exposed individual but also is passed on to the next generation, probably via the paternal lineage. As such, BAT plays a role in acute, chronic, and transgenerational adaptation to cold environment in humans.

## Introduction

Homeothermic mammals keep their body temperature constant at ambient temperatures lower than the thermoneutral zone, by the various physiological responses including vasoconstriction of the skin to reduce heat loss and skeletal muscle shivering to increase heat production. In addition to shivering thermogenesis, mammals have autonomically regulated non-shivering thermogenic mechanisms. A site of non-shivering thermogenesis (NST) is brown adipose tissue (BAT), a tissue typically found in hibernators and rodents with small body size. When they are exposed to cold, cold information is perceived by temperature sensors, transient receptor potential (TRP) channels on sensory neurons on the body surface, transmitted to the brain, and increases the activity of sympathetic nerves. Noradrenaline released from nerve endings stimulates brown adipocytes via the *β*-adrenergic receptor and triggers intracellular events including hydrolysis of triglyceride, oxidation of resulting fatty acids, and activates uncoupling protein 1 (UCP1), a thermogenic molecule specific to brown adipocyte mitochondria [1, 2].

When animals are exposed to cold for a long time, they adapt to their surroundings by increasing the number of brown adipocytes [3]. In addition to BAT hyperplasia, prolonged cold exposure gives rise to an apparent induction of UCP1-positive adipocytes in white adipose tissue. This type of adipocytes, termed “beige” or “brite” cells, is developmentally distinct from “classical” brown adipocytes [4, 5] and has the thermogenic potential. Thus, chronic cold exposure results in increased capacity of NST through increasing brown adipocyte number and induction of beige adipocyte.

The presence of BAT in humans was first demonstrated by Hatai, who found the multilocular brown-like adipocyte in the tissue obtained from the interscapular and neck regions of human fetuses [6]. The following anatomical studies, however, reported that BAT in humans was present in considerable amounts in fetuses and infants [7], but disappeared rapidly during postnatal periods, and was difficult to be macroscopically identified in adults [8, 9], except in patients with a catecholamine producing tumor, pheochromocytoma [1]. Accordingly, it had been a general contention that BAT is absent or of minute amounts and plays negligible roles in healthy adult humans [10]. However, this conventional view was challenged by studies using a radionuclide technique for diagnosis of cancer, fluorodeoxyglucose (FDG)-positron emission tomography (PET), and computed tomography (CT), which demonstrated the existence of considerable amounts of metabolically active BAT in adult humans [10–14]. The abundant mRNA and protein expressions of marker molecules, such as UCP1, in PET-detected BAT depots were confirmed by gene expression analysis and immunohistochemistry in biopsy and autopsy samples [11–14]. The rediscovery of human BAT has greatly accelerated preclinical and clinical studies on BAT. In this review, we summarize our current understanding on human BAT, with special reference to its thermogenic activity and roles in cold adaptation.

### Cold-induced activation of human BAT

As mentioned above, cold exposure activates triglyceride hydrolysis and oxidation of resulting fatty acids, which are indispensable for supplying energy source of thermogenesis. Studies in small rodents have reported that cold exposure also activates glucose utilization in BAT [15, 16], as reflected by tissue 2-deoxyglucose (2-DG) uptake, for a sufficient supply of oxaloacetate to enable the rapid oxidation of fatty acids and acetyl coenzyme A, and also for recovery of cellular adenosine triphosphate levels by activating anaerobic glycolysis. The stimulatory effects of cold exposure on 2-DG uptake are mimicked by *β*-adrenergic agonist administration but abolished by *β*-adrenergic blockade or UCP1-deficient animals [16, 17]. Thus, cold-induced 2-DG uptake into BAT is closely linked to UCP1-dependent thermogenesis.

In humans, tissue glucose utilization can be examined by the PET imaging technique using ^18^F-labelled 2-DG (FDG) as a tracer. Clinically, the FDG-PET technique has been used for diagnosis of malignant tumor, but it sometimes detects symmetrical FDG uptake into non-tumor adipose tissue in the shoulder and thoracic vertebral regions [18]. Such FDG uptake is increased in colder seasons [19] and reduced by pretreatment with *β*-adrenergic blockers [20]. We confirmed these clinical observations by dedicated studies in healthy volunteers [11]: that is, participants were overnight fasted and kept in a room at 28 °C (warm condition) or under a mild cold condition at 19 °C with light clothes. One hour later, they were injected with FDG intravenously, kept for another 1 h, and underwent whole-body FDG-PET examination. Under the warm condition, FDG uptake was found in the brain, heart, and marginally in the liver but undetectably in adipose tissue. When the same subjects were kept under the cold condition for 2 h, a clear and intense FDG uptake was found in adipose tissue at the supraclavicular and paravertebral regions, in addition to the brain and heart. No notable FDG uptake was found in skeletal muscle and adipose tissue at other regions. Under such a mild cold condition, muscle shivering was undetected at least during the 2-h period. Similar stimulatory effects of cold exposure on FDG uptake were reported by several research groups [12, 14, 21–26], although the condition of cold exposure was different between the groups (Table 1). Moreover, *β*-adrenergic agonist administration was reported to evoke comparable FDG uptake with those induced by cold exposure [24]. These results, together with those in forementioned animal studies, indicate the presence of BAT metabolically activated by acute cold exposure. This was further supported by histological and molecular biological observations, demonstrating the presence of numerous multilocular adipocytes expressing UCP1 mRNA/protein in fat pad samples obtained from the supraclavicular region [11, 12, 14]. Cold-induced metabolic activation of human BAT is also confirmed by PET using ^18^F-labelled fatty acid derivatives, [^18^F]fluoro- 6-thia-heptadecanoic acid, and ^15^O-labelled oxygen gas and water [22, 25, 27, 28].

**Table 1:** Effect of acute cold exposure on BAT and cold-induced non-shivering thermogenesis

Participant			Condition	BAT activity	CIT (kcal/day)			Reference
N (M/F)	Age	BMI			Low BAT	High BAT	Difference	
13 (13/0)	22.8	20.7	19 °C, light clothing, foot on ice, 2 h	- ~ + + +	42	410	368	Yoneshiro et al. Obeisty 2011 (ref. [36])
27 (7/20)	40.2	22.8	17 °C, light clothing, 2 h	- ~ + + +	167	287	120	Orava et al. Cell Metab 2011 (ref. [21])
6 (6/0)	23 to 42	23.7 to 31.0	19 °C cooling suit, 3 h	- ~ + + + +	2002 (77% of EE), shivering			Ouellet et al. J Clin Invest 2012 (ref. [22])
51 (51/0)	24.4	22.0	19 °C, light clothing, 2 h	- ~ + + +	78	252	174	Yoneshiro et al. J Clin Invest 2013 (ref. [44])
25 (10/15)	30.8	23.8	15.5 °C, light clothing, 2.5 h	- ~ + + +	7	237	230	Muzik et al. J Nucl Med 2013 (ref. [25])
24 (14/10)	28.1	20 to 27	19 °C, 12 h	- ~ + +	88 (5% of EE)			Chen et al. JCEM 2013 (ref. [40])
12 (12/0)	24	25.5	18 °C cooling suit, 3 h	- ~ + + + +	1886 (82% of EE), shivering			Blondin et al. J Physiol 2015 (ref. [26])
15 (15/0)	22.2	22.7	14 °C cooling suit, 2 h	- ~ + + +	128			Cypess et al. Cell Metab 2015 (ref. [24])
18 (18/0)	21.8	20.5	19 °C, light clothing, 2 h	- ~ + + +	120	256	136	Wakabayashi et al. EJAP 2020 (ref. [41])

-Undetectably low, + high

During the last 18 years starting from 2006, we have collected a total of 621 FDG-PET data from 583 healthy Japanese volunteers aged 18 ~ 73 years (487 males and 96 females). Our results, together with those of other groups, as tentatively summarized in some review articles [5, 29–31], demonstrate that human BAT activated by cold exposure is frequently detected in the supraclavicular and paravertebral regions and sometimes in the axillary and cervical regions. There is a marked individual variation in the activity/amount, and some participants show no detectable BAT even after cold exposure. The prevalence of participants with detectable BAT decreases with age, being about two-third in the 20 s but only one-twentieth in the 50 s [32]. The activity/amount inversely relates to body fatness [10, 11, 32]. It shows seasonal variations, being higher in winter [11]. Based on these results, in the next sections, we will discuss the pathophysiological roles of human BAT in NST, energy homeostasis, body temperature regulation, and cold adaptation. As the UCP1-positive adipose depot consists of a mixture of brown and beige adipocytes in humans [33, 34], hereafter, we shall refer to it collectively as BAT, unless otherwise specified.

### Cold-induced NST and BAT

Although FDG-PET/CT is useful for visualizing and evaluating BAT in humans [35], total heat production and energy consumption in BAT cannot be estimated from the FDG uptake because it also utilizes other energy substrates [22, 26]. Moreover, insulin administration causes a prompt and remarkable increase in FDG uptake into BAT independently of UCP1 activation nor heat production [21]. However, as discussed below, several lines of evidence support that human BAT detected by FDG-PET/CT has actually a thermogenic potential, thereby playing a role in NST and whole-body energy homeostasis.

The tissue oxidative metabolism and thermogenesis can be quantified directly by measuring tissue oxygen extraction and blood flow. Using ^15^O[O_2_]-, 1^5^O[CO]-, and ^15^O[H_2_O]-PET, two research groups reported that acute mild cold exposure evoked a marked increase in oxygen consumption by cervical-supraclavicular adipose tissue, in parallel with increased whole-body energy expenditure (EE), in healthy subjects having large amounts of BAT but only slightly in those lacking BAT [25, 27]. These results clearly indicate that human BAT has actually a thermogenic potential.

Direct monitoring of the temperature of the skin (*T*_skin_) overlying BAT depots, by using a wireless thermistor probe and an infrared thermography camera, is another approach for semiquantitative assessment of BAT thermogenic activity. In fact, several studies have demonstrated that cold-induced changes in skin temperature of the supraclavicular region close to BAT depots, but not in that of a control chest region, positively correlated with the activity/volume of BAT estimated by FDG-PET [36–39]. Such a region-specific response in skin temperature would be attributable to BAT thermogenesis.

Possible contribution of BAT to whole-body EE, particularly to NST, has been tested by examining the relationship between cold-induced thermogenesis (CIT) and BAT activity/volume estimated by FDG-PET. We [36] first reported in young healthy volunteers that EE under a warm condition at 27 °C was largely dependent on body size and comparable in those having and lacking active BAT. When they were exposed to cold at 19 °C for 2 h, EE increased by 410 kcal/day (28% of EE at 27 °C) in those having BAT but only 42 kcal/day (3%) in those lacking BAT. Muscle shivering was not observed at least for the 2-h period of cold exposure. Therefore, the difference in cold-induced change in EE, i.e., CIT, between participants having and lacking BAT would be attributable to EE by BAT activation. This was further supported by a finding of a highly positive correlation between CIT and the BAT activity quantified from FDG uptake into supraclavicular fat tissue. Similar results were also reported by several research groups, showing BAT-associated CIT is 120–370 kcal/day, 15–25% of EE under warm conditions (Table 1). Chen et al. [40] examined the effects of exposure to minimal cold, well within the range of climate-controlled buildings, and found that even such milder cold exposure evoked slight but significant increases in EE (5%) and BAT activity (31%). As such, exposure to mild cold increases whole-body EE, in association with increased BAT activity, without notable muscle shivering, suggesting a contribution of BAT to CIT.

Some groups [22, 26] reported much larger CIT of about 2000 kcal/day, ~80% of EE under warm conditions (Table 1). Such higher CIT is largely due to stronger cold exposure, which induces muscle shivering and masks BAT-associated thermogenesis. Wakabayashi et al. [41] measured whole-body EE and pectoralis major muscle activity in young participants, while the room temperature was successively lowered from 28 to 18.6 °C and to 11.6 °C. At 18.6 °C, participants with low BAT activity showed a lower CIT than those with high BAT activity, but at 11.6 °C, they began shivering earlier and more intensively, and EE eventually rose to comparative high levels in the two participant groups. Thus, heat production in cold conditions is first preceded by NST by BAT, followed by skeletal muscle shivering, the two working together to maintain body temperature.

As discussed above, the idea of a significant role of BAT in CIT seems well accepted at present [42], but there are some reports against this consensus [25, 26]: that is, when the tissue oxidative metabolism and thermogenesis are directly quantified by measuring tissue oxygen extraction and blood flow. Using ^15^O[O_2_]-, ^15^O[CO]-, and ^15^O[H_2_O]-PET, cold-induced BAT thermogenesis is only ~ 25 kcal/day, being much lower than the whole-body CIT shown in Table 1. The precise reason of such conflicting observations is unknown at present, but it may be possible that they may underestimate the whole-body total BAT mass [30]. Alternatively, thermogenesis in some tissues other than BAT may be more important in cold-induced NST. Further studies are needed to clarify the physiological mechanisms of cold-induced NST.

### BAT and cold adaptation

The regulation of BAT function differs between acute and chronic activation. Prolonged cold exposure results in a gradual decrease in shivering, while whole-body EE remains elevated, implying an increased NST by BAT [43]. In fact, animal studies have showed prolonged cold exposure recruits thermogenic brown and beige adipocytes, thereby increasing NST capacity [3–5]. Similar adaptation to cold was also demonstrated in humans. We [44] reported that daily cold exposure of 2 h per day at 17 °C for 6 weeks resulted in an increase in CIT accompanied with an increase in BAT activity and a significant decrease in body fat mass. Interestingly, changes in BAT activity and body fat mass were negatively correlated, confirming a role of BAT in the regulation of body fatness. Increased BAT activity/volume after repeated cold exposure was also reported by several other studies (Table 2). In a crossover study of 4-month duration, consisting of four consecutive blocks of 1-month overnight temperature acclimation (24–19 °C to 24–27 °C), Lee et al. [45] demonstrated that monthly acclimation modulated BAT reversibly, enhancing and suppressing its abundance and activity in mild cold and warm conditions, respectively. Van der Lans et al. [46] reported increased NST and BAT activity after a relatively short period of repeated cold exposure (15 °C, 6 h/day, 10 days), with a delayed onset of shivering. Such a short-term cold acclimation recruits BAT and increases NST in obese people [47] and type 2 diabetic patients [48]. Blondin et al. [49, 50] also reported a substantial increase in BAT activity accompanied by a modest decrease in shivering intensity after 4-week cold acclimation, being consistent with a report by Davis [43]. These results seem in line with a study in indigenous people in Siberia, demonstrating higher UCP1 expression in fat cells obtained from outdoor workers than indoor workers [51]. In a meta-analysis, Huang et al. [52] reported that the prevalence of activated BAT decreased by 1% for each 5 °C increases in average outdoor temperature.

**Table 2:** Effect of chronic cold exposure on BAT and cold-induced non-shivering thermogenesis

Participants			Condition	Increase in BAT activity/volume	CIT (kcal/day)		Reference
N (M/F)	Age	BMI			Before	After	
12 (12/0)	26.3	23.8	17 °C, 2 h/day, 6 weeks	58%	108	289	Yoneshiro et al. J Clin Invest 2013 (ref. [44])
17 (8/9)	23	21.6	15–16 °C, 2–6 h/day, 10 days	35%	206	275	van der Lans et al. J Clin Invest 2013 (ref. [46])
5 (5/0)	21	22	19 °C, 24 h/day, 4 weeks	54%	152	177	Lee et al. Diabetes 2014 (ref. [45])
8 (8/0)	59.3	29.8	14–15 °C, 2–6 h/day, 10 days	57%	18	113	Hanssen et al. Nat Med 2015 (ref. [48])
10 (10/0)	36	32.9	14–15 °C, 2–6 h/day, 10 days	26%	207	275	Hanssen et al. Diabetes 2016 (ref. [47])
9 (9/0)	23	24.4	10 °C cooling suit, 2 h/day, 4 weeks	182%, vol. 45%	3727a	3652a	Blondin et al. J Physiol 2017 (ref. [50])

^a^Shivering thermogenesis + NST + basal EE

It is thus obvious that adaption to cold environment is closely associated with increased capacity of BAT thermogenesis in humans. This provides a plausible explanation for the seasonal variation in BAT activity and NST. In small body hibernating mammals, the amount of BAT shows a clear seasonal variation, being high in winter for arousal from hibernation. In humans, retrospective readings of FDG-PET in thousands of patients have revealed that BAT activity/prevalence shows marked seasonal variations, being associated with ambient temperature, thereby low in summer and high in winter in northern hemisphere countries [53–55]. In a prospective study in healthy participants, we [11] found clear seasonal variations of cold-induced activation of BAT: that is, almost all participants showed very low BAT activities in summer but much increased activities when they underwent FDG-PET again in winter. Similar seasonal changes in the activity and volume of BAT were also reported in other prospective studies [56–58]. We [59] also found that CIT significantly increased in winter compared with summer, being greater in participants with high BAT activity than those with low BAT activity. The cold-induced drop of tympanic temperature and *T*_skin_ in the supraclavicular region close to BAT deposits were smaller in the high BAT group than in the low BAT group in winter, but not in summer. In contrast, the drop of *T*_skin_ in the subclavicular and peripheral regions distant from BAT was similar in the two groups in both seasons. These results, together with the previously reported seasonal variations in CIT [60, 61], indicate a role of BAT in seasonal changes in the thermogenic and thermal responses to cold exposure in humans. Cold adaptation of BAT is thus involved in seasonal variations of CIT, thereby in fine regulation of body temperature throughout a year.

### Parental cold exposure and offspring BAT

The fact that BAT mass/activity shows seasonal fluctuations implies that the inducible effects of cold stress are reversible. In fact, the increased NST and BAT activity induced by repeated cold exposure decrease to basal levels several weeks after stopping daily cold exposure [45]. In contrast, environmental stress including cold exposure during early-life stages might modify the postnatal development of tissue functionality, thereby leading long-lasting metabolic changes in adulthood. This idea is known as the theory of Developmental Origin of Health and Disease (DOHaD), which is typically demonstrated by the effects of nutritional environment during the prenatal, neonatal, and early postnatal periods on metabolic phenotypes of the offspring adulthood through epigenetic control mechanisms. For example, maternal obesity induced by high-fat diet feeding before and throughout gestation and lactation leads to metabolic dysfunction such as obesity, glucose intolerance, and impaired BAT thermogenesis in offspring [62–66]. With regard to cold stress, there have been reports that rearing newborn animals at lower environmental temperatures improves their tolerance on subsequent exposure to cold accompanied with increased BAT activity in young adults [67–70]. In humans, Levy et al. [71] examined retrospectively the effects of cold exposure during gestation infancy, early childhood, middle childhood, and adolescence on adult BAT thermogenesis and suggested early childhood as a sensitive stage for developmental plasticity of BAT. However, there have been no prospective studies, as far as we know, examining the effects of cold exposure during early childhood on BAT and NST in adulthood.

Some animal studies have suggested that cold stress influences thermoregulation and metabolism not only in the exposed individual but also in the next generation. Symons et al. [72] reported that maternal cold exposure during late pregnancy resulted in a greater CIT and an increased thermogenic activity of BAT in the neonatal lamb. To analyze possible effects of gestational ambient temperature on fetal programming, Oelkrug et al. [73] kept female mice at different ambient temperatures (18, 23, and 30 °C) for several weeks before and during pregnancy. Maternal body weight gain did not differ during pregnancy, and analysis at the gestational day of 18 showed higher thermogenic activity of BAT in those kept at lower ambient temperature, being consistent with the effects of chronic cold exposure in nonpregnant animals. Immediately after birth, pups were housed at a conventional housing temperature of 23 °C and analyzed at the age of 2–3 months. Offsprings from dams kept at lower ambient temperature showed lower muscle and lean body masses, lower whole-body EE, and impaired glucose tolerance. Thermogenic activity of BAT, however, did not differ between offsprings from dams kept at the three ambient temperatures. No notable effect of maternal cold exposure on offspring BAT was also reported in mice [74]. The DOHaD theory acknowledges that environmental stress during pregnancy or lactation triggers predictive adaptation during embryonic and postnatal development via the maternal lineage. It remained to be debated, however, whether maternal cold exposure has an impact on the development of offspring BAT in humans.

Recently, evidence has been accumulated for a link between preconception stress and the phenotypes of the offspring via the paternal linkage [75, 76]. Sun et al. [74] demonstrated in mice that cold exposure of males, but not females, before mating resulted in increased BAT, improved systemic metabolism, and protection from diet-induced obesity of the male offspring. They suggested that cold exposure induces an epigenetic programming including alteration of DNA methylation of the sperm such that the offspring equips active BAT and an improved adaptability to cold environment. In line with these findings in mice, they showed in humans that individuals with active BAT were more likely to have been conceived in the colder months, whereas those without active BAT were in the warmer months. We also examined the association of cold-activated BAT with the seasons of fertilization or birth in young healthy Japanese adult volunteers [77]. We found that BAT activity was higher in individuals from mothers conceived in the cold season than in those conceived in the warm season, while it was indistinguishable in the individuals born in either season. A similar seasonal difference was also found in BAT-associated NST, being higher in the cold fertilization group. A detailed analysis revealed that outdoor temperature and daily temperature variation before conception are the key meteorological factors for the transgenerational control of BAT. This is quite compatible with the forementioned findings in mice, suggesting transgenerational regulation of human BAT may originate from the paternal lineage. These results indicate that the metabolic fate of BAT in adulthood is predetermined by parental exposure to meteorological environments during the period around fertilization in healthy humans. Research into the epigenetic mechanisms of the transgenerational regulation is currently underway, particularly focusing on DNA methylation of sperm and noncoding RNA in seminal fluid.

In addition to the epigenetic mechanisms, various genes are intimately involved in the regulation of human BAT. We reported that single-nucleotide polymorphism (SNP) in the *UCP1* and *ADRB3,* that encodes *β3* adrenergic receptor, genes have a weak but significant effect on age-related changes in BAT [78]. Nishimura et al. also showed UCP1 genotype changes alter the efficiency of cold-induced NST [79]. Moreover, recent our studies revealed an impact of single-nucleotide changes in the *ADRB2*, that encodes *﻿β*2 adrenergic receptor, and *LEPR*, that encodes leptin receptor, genes on BAT activity [80, 81]. The effect of SNPs could explain, at least in part, marked individual and ethnic differences in BAT activity [82]. It is also likely that various BAT-related gene SNPs are involved in the natural selection of cold adaptability during migration to and colonization in colder regions [81, 83, 84].

## Conclusion

**Fig. 1:**
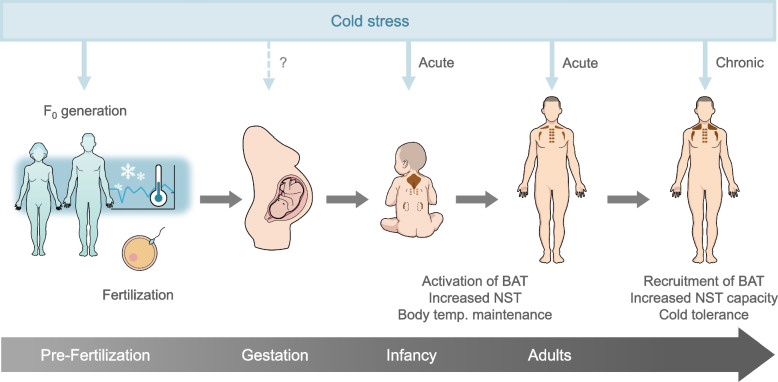
Pre- and postnatal effects of cold stress on BAT and non-shivering thermogenesis. In infancy and adulthood, acute cold exposure activates BAT and increases NST to prevent hypothermia. Prolonged cold exposure recruits BAT, leading to increased capacity of NST and improved cold tolerance. BAT-associated NST capacity in adulthood is also increased by parental cold exposure during the period around fertilization. Modified from Yoneshiro et al. [77]

Cold stress influences thermogenic BAT in various time spans (Fig. 1). Acute cold exposure activates BAT to induce NST in couples of minutes, which contributes to immediate maintenance of body temperature. Prolonged cold exposure recruits BAT by proliferating brown adipocyte and inducing thermogenic beige adipocyte, leading to increased capacity of NST and improved cold tolerance. Such BAT adaptation not only occurs in the exposed individual but also is passed on to the next generation. As such, BAT plays a role in acute, chronic, and transgenerational adaptation to cold environment in humans.

Despite the remarkable advances in human BAT researches, there are some critical problems, particularly on the method to assess human BAT. As noted in the previous sections, to date, FDG-PET/CT is used as a gold standard tool; however, this has serious limitations, including the enormous cost of devices and radiation exposure, which make frequent and repeated measurements difficult. To promote prospective human studies, it is an urgent need to establish less invasive, less expensive, and simpler methods for quantitative assessment of human BAT.

## Data Availability

Not applicable.

## Abbreviations

BAT: Brown adipose tissue
CIT: Cold-induced thermogenesis
CT: Computed tomography
2-DG: 2-Dexoyglucose
EE: Energy expenditure
FDG: Fluorodeoxyglucose
NST: Non-shivering thermogenesis
PET: Positron emission tomography
Tskin: Skin temperature
TRP: Transient receptor potential
UCP1: Uncoupling protein 1
